# Hydrogen bonding patterns and C—H⋯π inter­actions in the structure of the antiparkinsonian drug (*R*)-rasagiline mesylate determined using laboratory and synchrotron X-ray powder diffraction data

**DOI:** 10.1107/S2052520623007758

**Published:** 2023-10-11

**Authors:** Analio J. Dugarte-Dugarte, Robert A. Toro, Jacco van de Streek, José Antonio Henao, Andrew N. Fitch, Catherine Dejoie, José Miguel Delgado, Graciela Díaz de Delgado

**Affiliations:** a Laboratorio de Cristalografía-LNDRX, Departamento de Química, Facultad de Ciencias, Universidad de Los Andes, Mérida, 5101, Venezuela; b Grupo de Investigación en Química Estructural (GIQUE), Escuela de Química, Facultad de Ciencias, Universidad Industrial de Santander, Bucaramanga, Colombia; c Avant-garde Materials Simulation, Alte Str. 2, Merzhausen, D-79249, Germany; d European Synchrotron Radiation Facility (ESRF), 71 Avenue des Martyrs, CS40220, Grenoble, Cedex 9 38043, France; University of Geneva, Switzerland

**Keywords:** crystal structure, rasagiline mesylate, structure determination, synchrotron radiation, DFT-D calculations, Hirshfeld surface analysis, Parkinson’s disease

## Abstract

The structure of (*R*)-rasagiline mesylate [(*R*)-RasH^+^·Mes^−^], an active pharmaceutical ingredient used to treat Parkinson’s disease, is presented. The structure was determined from laboratory and synchrotron powder diffraction data, refined using the Rietveld method, and validated and optimized using dispersion-corrected DFT calculations. The unit-cell parameters obtained in both experiments are in good agreement and the refinement with both datasets converged to good agreement factors. The final parameters obtained from laboratory data were *a* = 5.4905 (8), *b* = 6.536 (2), *c* = 38.953 (3) Å, *V* = 1398.0 (4) Å^3^ and from synchrotron powder data were *a* = 5.487530 (10) Å, *b* = 6.528939 (12) Å, *c* = 38.94313 (9) Å, *V* = 1395.245 (5) Å^3^ with *Z* = 4 and space group *P*2_1_2_1_2_1_. Preferred orientation was properly accounted for using the synchrotron radiation data, leading to a March–Dollase parameter of 1.140 (1) instead of the 0.642 (1) value obtained from laboratory data. In the structure, (*R*)-RasH^+^ moieties form layers parallel to the *ab* plane connected by mesylate ions through N—H⋯O and C—H⋯O hydrogen bonds. These layers stack along the *c* axis and are further connected by C—H⋯π interactions. Hirshfeld surface analysis and fingerprint plot calculations indicate that the main interactions are: H⋯H (50.9%), H⋯C/C⋯H (27.1%) and H⋯O/O⋯H (21.1%).

## Abstract 2

Extensive hydrogen bonding and C—H⋯π interactions dominate the packing of molecules in the antiparkinsonian drug (*R*)-rasagiline mesylate. The structure was determined from laboratory and synchrotron powder diffraction data and validated using DFT-D calculations.

## 1. Introduction

**Figure 1:**
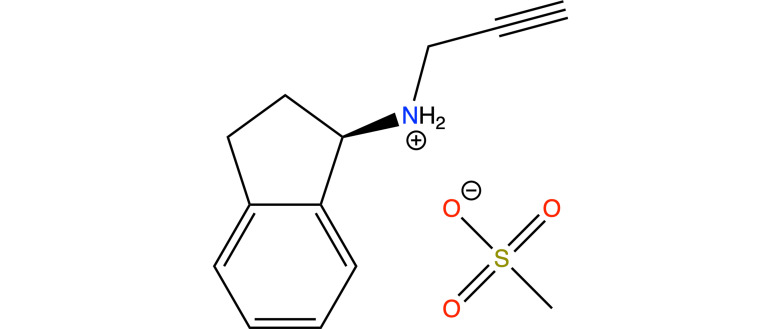
Molecular diagram for (*R*)-RasH^+^·Mes^−^.

Rasagiline [(*R*)-*N*-(prop-2-yn-1-yl)-2,3-di­hydro-1*H*-inden-1-amine, C_12_H_13_N, see Fig. 1 ▸] is an oral, second-generation, selective, irreversible mono­amine oxidase B (MAO-B) inhibitor used for the treatment of Parkinson’s disease and as an adjunct therapy to levodopa in more advanced cases, approved in the USA and EU. It also showed neuroprotective and neurorescue properties (McCormack, 2014 ▸; Szökő *et al.*, 2018 ▸; Nagai & Hattori, 2020 ▸). It is marketed under the name Azilect as its mesylate salt form [C_12_H_14_N^+^·CH_3_SO_3_^−^, (*R*)-RasH^+^·Mes^−^]. Rasagiline is also formulated as a hemitartrate derivative. Other rasagiline salts have proven to be pharmaceutically acceptable but some of them have problems in drug manufacturing because they cannot be directly compressed into tablets due to their hygroscopicity (Stahl, 2008 ▸). Rasagiline is five- to tenfold more potent than selegiline, also known as l-deprenyl (Tábi *et al.*, 2020 ▸), the first MAO-B inhibitor and a well established antiparkinsonian drug for more than 40 years. Another advantage of rasagiline over selegiline is that it does not metabolize to amphetamines and does not display sympathomimetic and neurological effects (Zhou *et al.*, 2018 ▸).

A search in the Cambridge Structural Database (CSD, version 2022.2.0) (Groom *et al.*, 2016 ▸) using the molecular moiety of rasagiline leads to only one report. It corresponds to the crystal structure of rasagiline ethane­disulfonate, reference code NOJKON (Brüning *et al.*, 2008 ▸). A similar search and a search by name in the ICDD PDF-4/organics database (Gates-Rector & Blanton, 2019 ▸) produced two reports. One of them (PDF 00-066-0982) contains the peak positions of the unindexed pattern of rasagiline mesylate reported in a patent (Thanedar *et al.*, 2011 ▸). The other entry (PDF 02-098-3238) contains the calculated pattern of rasagiline ethane­disulfonate, based on the structural parameters contained in the NOJKON CSD entry.

It must be mentioned that a conformational and vibrational study of rasagiline using density functional theory [DFT/ B3LYP/6-31++G(d,p)] calculations has been reported by Başköse *et al.* (2012 ▸). This study involved the free base and its mesylate and ethane­disulfonate derivatives.

After searching for ‘rasagiline mesylate’ on the Google Patents site (https://patents.google.com), many entries are encountered. Some of them refer to the preparation of two crystal forms of rasagiline mesylate: form I (Masllorens-Llinas & Duran-Lopez, 2011 ▸; Gore *et al.*, 2010 ▸) and form II (Youdim *et al.*, 1996 ▸; Dwivedi *et al.*, 2012 ▸; Sathe *et al.*, 2014 ▸). They show experimental powder diffraction patterns, but no structural information is reported.

Within a project of the Grant-in-Aid program supported by the International Centre for Diffraction Data (ICDD) intended to register high-quality X-ray powder diffraction data of pharmaceutical materials of interest with none or limited structural information in the literature, several compounds of pharmaceutical and general chemical interest have been studied in our laboratory (Dugarte-Dugarte *et al.*, 2021 ▸, 2022 ▸; Toro *et al.*, 2022 ▸). Since there is no structural information on rasagiline mesylate, it was decided to record its powder pattern and undertake the structure determination of this compound initially from laboratory data. The structural model obtained was validated by dispersion-corrected DFT calculations. The material under study was also characterized by ATR-IR and TGA/DSC. Since the structure refinement showed strong preferred orientation, indicated by a rather low March–Dollase parameter, synchrotron data were used for the final refinement. Hirshfeld surface analysis and fingerprint plot calculations provide additional insights into the role that different intermolecular interactions play in the packing arrangement of this important compound.

## 2. Experimental

### 2.1. Powder diffraction pattern

Initially, a small portion of the sample, provided by Tecnoquímicas (Cali, Colombia), was ground, then mounted on a zero-background holder. The X-ray powder diffraction data were registered at room temperature with a BRUKER D8 ADVANCE diffractometer working in Bragg–Brentano geometry. This instrument is equipped with a Cu *K*α source, working at 40 kV and 30 mA, and a LynxEye detector. The pattern was recorded from 4.00 to 70.00° in steps of 0.02° (2θ) at one second per step. The standard instrument settings (Ni filter of 0.02 mm, primary and secondary Soller slits of 2.5°, a divergence slit of 0.2 mm, scatter screen height of 3 mm) were used.

High-resolution X-ray powder diffraction data were collected at beamline ID22 at ESRF, Grenoble, as part of a LAAAMP FAST Team award. A carefully ground sample of the rasagiline mesylate was used to fill a 1.0 mm thin-walled borosilicate capillary. Data were collected at ambient temperature with a wavelength of 0.35417581 Å between −10 and 40.000° in 2θ (continuous scan rate of 20° min^−1^, data sampling intervals of 0.0005°). The capillary was spun at 919 rpm and translated by 1.2 mm between scans to expose a fresh region of the sample to minimize possible radiation damage. Scans were collected at 31 positions along the capillary. Although the irradiated sections of the sample experienced darkening after exposure to the beam, ATR-IR spectra recorded on these sections did not indicate reaction or decomposition of the sample. The scans were checked for consistency then all summed together into steps of 0.001°, taking account of the angular offsets between the 13 channels of detection, their relative efficiencies and the evolution of the ring current during the course of the measurement (Wright *et al.*, 2003 ▸). Corrections to 2θ values were also made for the effects of axial divergence, filtering axially on an estimated broadening due to the curvature of the Debye–Scherrer cones of 0.003° (Fitch & Dejoie, 2021 ▸). The analysis used the summed data in the 2θ range 0.5–20°.

### 2.2. Spectroscopic and thermal analysis

ATR-FTIR spectra were recorded on a FTIR Bruker Tensor 27 spectrophotometer coupled to a Bruker platinum ATR cell. TGA and DSC measurements were carried out simultaneously on a NETZSCH STA 449 F3 Jupiter apparatus under a dynamic nitro­gen atmosphere (50 ml min^−1^ N_2_ flow) in the temperature range 25 to 500°C, heating at a rate of 10°C min^−1^.

### 2.3. Pattern indexing, structure determination and refinement.

The indexing of the patterns was carried out with *DICVOL14* (Louër & Boultif, 2014 ▸) as implemented within *PreDICT* (Blanton *et al.*, 2019 ▸) and with *CONOGRAPH* (Esmaeili *et al.*, 2017 ▸). The Pawley fit of the patterns was accomplished with *TOPAS-Academic* (Coelho, 2018 ▸). *DASH 4.0.0* (David *et al.*, 2006 ▸), *CONOGRAPH* and *DAJUST* (Vallcorba *et al.*, 2012 ▸) were used in the analysis of reflection conditions to obtain possible space groups. *DASH 4.0.0* was also used to determine the structure and the refinement was carried out with *TOPAS-Academic*. The analysis of bond lengths and angles, torsion angles, hydrogen bonding, intermolecular contacts, and packing arrangement was performed with *PLATON* (Spek, 2020 ▸), *Mercury* (Macrae *et al.*, 2020 ▸) and *Mogul* (Bruno *et al.*, 2004 ▸). Graphical representations were prepared with *Mercury* and *DIAMOND* (Brandenburg, 1999 ▸). *enCIFer* (Allen *et al.*, 2004 ▸) and *publCIF* (Westrip, 2010 ▸) were used for CIFs and manuscript preparation and editing. Crystal data, data collection and structure refinement details are summarized in Table 1 ▸.

## 3. Computational studies

### 3.1. Theoretical calculations

With the use of *Mercury*, ten rotamers of (*R*)-RasH^+^ were generated, applying the following parameters: a maximum of 500 conformations, a maximum of four unusual rotamers, with 0.10 minimum rotamer probability for rotamer creation. Next, all the rotamers were optimized with PM7 geometry optimization in *Mopac 2022* (Stewart, 2013 ▸, 2022 ▸). Then, each model was optimized using the B3LYP/6-311G(d,p) DFT formalism (Hohenberg & Kohn, 1964 ▸; Krishnan *et al.*, 1980 ▸) in the gas phase. Density functional calculations were performed and the geometries of each previously pre-optimized rotamers of (*R*)-RasH^+^ were further optimized using the *GAMESS-US* (Barca *et al.*, 2020 ▸) program through the web-based job submission interface ChemCompute (https://chemcompute.org/) (Perri & Weber, 2014 ▸).

### 3.2. DFT-D calculations

The crystal structure obtained from XRPD was energy-minimized with the program *GRACE* (Neumann, 2019 ▸), which calls VASP (Kresse & Furthmüller, 1996 ▸) for single-point DFT calculations with the PBE functional (Perdew *et al.*, 1996 ▸) to which a dispersion correction (Neumann & Perrin, 2005 ▸) has been added. The method has been extensively validated against about 600 crystal structures and the upper limit for the root-mean-square Cartesian displacement between the structure from the Rietveld refinement, if correct, and the energy-minimized structure was established to be approximately 0.35 Å (van de Streek & Neumann, 2014 ▸). Details of the calculations can be found elsewhere (Neumann & Perrin, 2005 ▸).

### 3.3. Hirshfeld surface analysis

The software *CrystalExplorer21* (Spackman *et al.*, 2021 ▸) was used to produce fingerprint plots of the intermolecular interactions present in the structure determined in this work. The map of the parameter *d*_norm_ onto the Hirshfeld surface (Spackman & Jayatilaka, 2009 ▸) was calculated. This parameter is useful for visualizing the atoms involved in intermolecular contacts and the strength of such contacts.

## 4. Results and discussion

### 4.1. Laboratory data

The indexing of the recorded pattern carried out with *DICVOL14* (Louër & Boultif, 2014 ▸) as implemented in the *PreDICT* graphical user interface (Blanton *et al.*, 2019 ▸) using the first 20 peaks produced an orthorhombic unit cell. The analysis of all 71 diffraction maxima registered, performed with NBS*AIDS80 (Mighell *et al.*, 1981 ▸), using the unit cell obtained by *DICVOL14* led to the following unit-cell parameters: *a* = 5.4905 (8), *b* = 6.536 (2), *c* = 38.953 (3) Å, *V* = 1398.0 (4) Å^3^. The de Wolf (1968 ▸) and Smith–Snyder (Smith & Snyder, 1979 ▸) figures of merit obtained were *M*_20_ = 35.4 and *F*_30_ = 71.7 (0.0093, 45), respectively. The unit cell with the highest figures of merit, produced with *CONOGRAPH* (Esmaeili *et al.*, 2017 ▸; Oishi-Tomiyasu, 2013 ▸) using the first 25 peaks, was similar to the unit cell produced by *DICVOL14*. A reduced cell search in the CSD (Groom *et al.*, 2016 ▸) combined with a chemical elements search having only C, H, N, O and S yielded no hits.

**Figure 2:**
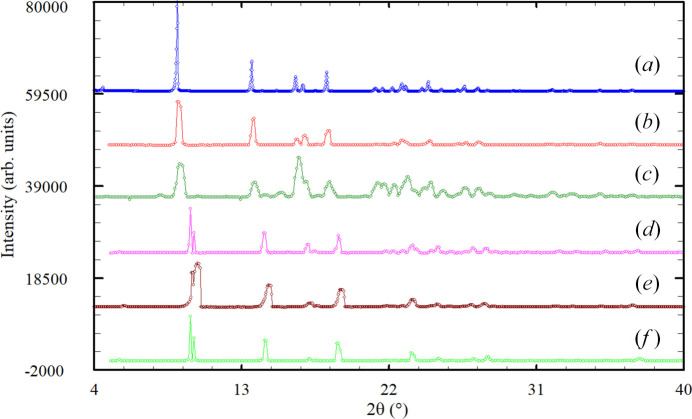
Comparison of the powder pattern recorded in the present study (*a*) for rasagiline mesylate [(*R*)-RasH^+^·Mes^−^] with the reported powder patterns of form I [(*b*) Masllorens-Llinas & Duran-Lopez (2011 ▸), (*c*) Gore *et al.* (2010 ▸)] and form II [(*d*) Youdim *et al.* (1996 ▸), (*e*) Dwivedi *et al.* (2012 ▸) and (*f*) Sathe *et al.* (2014 ▸)].

The superposition of the powder patterns reported in the patents for forms I (Masllorens-Llinas & Duran-Lopez, 2011 ▸; Gore *et al.*, 2010 ▸) and II (Youdim *et al.*, 1996 ▸; Dwivedi *et al.*, 2012 ▸; Sathe *et al.*, 2014 ▸) digitized using the on-line JADE Pattern Digitizer (ICDD, 2023 ▸; https://www.icdd.com/jade-pattern-digitizer) and the pattern recorded in the present work is shown in Fig. 2 ▸. The patterns of forms I and II look different. The powder patterns of form I are similar to the pattern recorded, indicating that it is the same phase.

The fit of the pattern was carried out with the Pawley algorithm by modelling the background, sample displacement errors, unit-cell parameters, peak shape parameters (including anisotropic broadening), and absorption correction using *TOPAS-Academic* (Coelho, 2018 ▸). A 15-term Chebyshev polynomial was used to model the background. The intermediate Gaussian–Lorentzian function was employed with a correction for axial divergence as proposed by the program. The Pawley refinement produced a good fitting of all the diffraction maxima recorded with residuals *R*_p_ = 0.0437, *R*_wp_ = 0.0661 and GoF = 1.035, confirming the correctness of the unit cell and the single-phase nature of the material. The analysis of the reflection conditions implemented in the *CONOGRAPH* software suggests *P*2_1_2_1_2_1_ as a possible space group. This space group was also suggested by the Bayesian extinction symbol algorithm in *DASH 4.0.0* (David *et al.*, 2006 ▸) and by *DAJUST* (Vallcorba *et al.*, 2012 ▸).

The initial molecular model, introduced as mol files, was built from the CIF of the CSD entries NOJKON (Brüning *et al.*, 2008 ▸) and QEYNAM (Gaztañaga *et al.*, 2018 ▸) for the (*R*)-RasH^+^ cation and the Mes^−^ anion, respectively. With this model and the profile parameters obtained from the Pawley fit, the crystal structure was determined with *DASH 4.0.0* (David *et al.*, 2006 ▸). Initially, without considering preferred orientation, the fitting of the profile led to a χ^2^ = 55.183 and it was not possible to obtain a good structural model. Using the March–Dollase function as model for the preferred orientation in the (001) plane, the structure was determined satisfactorily with χ^2^ = 13.078 and a March–Dollase parameter of 0.622. The refinement of the structure carried out with *TOPAS-Academic* (Coelho, 2018 ▸), using the same preferred orientation model, produced a good fit with residuals *R*_p_ = 0.0643, *R*_wp_ = 0.0869 and GoF = 1.340.

In spite of the good value of the root-mean-square Cartesian displacement (RMSCD) of 0.193 obtained, there were a few discrepancies with the DFT-D model of this structure, resulting from *GRACE* (Neumann, 2019 ▸) even though the RMSCD value is below the limit of 0.35 Å which is considered the upper limit acceptable for correct structures determined from powder diffraction data (van de Streek & Neumann, 2014 ▸).

**Figure 3:**
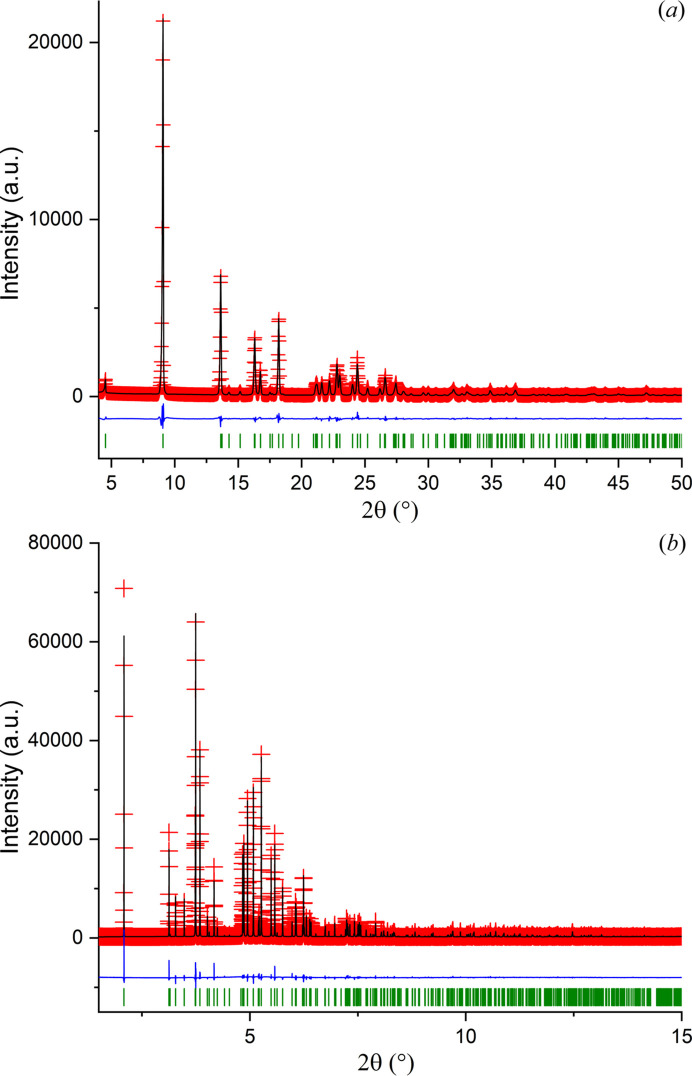
(*a*) Rietveld plot obtained after the structure refinement of (*R*)-RasH^+^·Mes^−^ using laboratory data and (*b*) Rietveld plot obtained after refinement using synchrotron data. The red crosses represent the experimental data. The black and blue lines represent the calculated pattern and the difference between the experimental and calculated profiles, respectively. The green vertical bars correspond to the positions of Bragg reflections.

The examination of the structure with *Mercury* (Macrae *et al.*, 2020 ▸) showed that the indane ring was planar, in contrast with the envelope conformation that it displays in the reported ethane disulfonate NOJKON (Brüning *et al.*, 2008 ▸). The refinement performed with *TOPAS-Academic* (Coelho, 2018 ▸), using the energy-minimized structure as the starting model, was very stable and proceeded smoothly. Fig. 3 ▸(*a*) shows the final Rietveld refinement plot. The refinement included an overall scale parameter, the background, the peak shapes (including anisotropic broadening), unit-cell parameters, atomic coordinates, four *B*_iso_ parameters, an absorption correction and a March–Dollase parameter. The bond distances and angles were restrained based on the values of the energy-minimized structure. Two planar restraints for the molecule with a standard deviation of 0.01 Å were also established. The isotropic atomic displacement parameters were constrained based on the type of atoms. The isotropic atomic displacement parameters for the hydrogen atoms were 1.2 times the parameter of the C or N atom to which they are attached.

In total, for the Cu *K*α data, 156 parameters were refined with 2261 data points (175 reflections), 99 restraints and four constraints. The final whole pattern fitting converged with good figures of merit: *R*_exp_ = 0.0649, *R*_p_ = 0.0613, *R*_wp_ = 0.0841, and GoF = 1.690. The March–Dollase parameter refined to 0.642 (1).

### 4.2. Synchrotron radiation data

The opportunity of collecting high-resolution synchrotron data in a capillary setting at the European Synchrotron Radiation Facility (ESRF), through a FAST Team award of the LAAAMP program, prompted us to carry out the structure refinement with synchrotron data to minimize the strong preferred orientation present in the pattern.

The refinement of the crystal structure of (*R*)-RasH^+^·Mes^−^ using synchrotron data included 125 parameters, 19501 data points (935 reflections) with 99 restraints and four constraints. The figures of merit for the fitting of the pattern converged to: *R*_exp_ = 0.0104, *R*_p_ = 0.07583, *R*_wp_ = 0.0812 and GoF = 7.814. As expected, the March–Dollase preferred orientation parameter improved substantially and refined to 1.140 (1). Fig. 3 ▸(*b*) shows the Rietveld refinement plot using the synchrotron radiation data. The very high GoF value of 7.814, equivalent to a χ^2^ value of 61.059, for an otherwise acceptable fit both in terms of the difference curve as well as the chemistry of the final model, indicates that the errors on the measured intensities as calculated assuming Poisson statistics are too optimistic and probably do not take into account some minor errors of a more systematic nature. The possibility of beam-induced damage cannot be ruled out.

**Figure 4:**
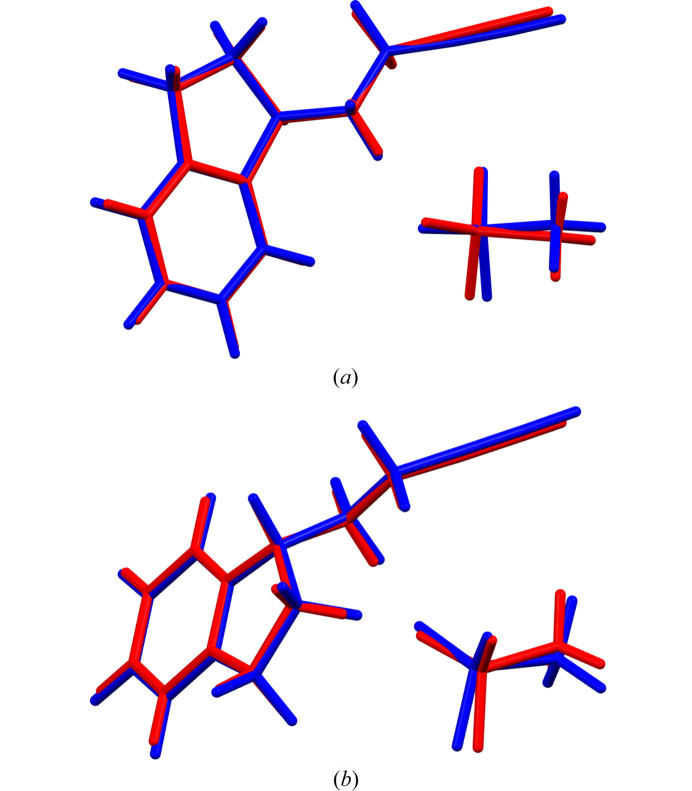
Superposition of the experimentally determined (red) and the energy-minimized (blue) structure for (*R*)-RasH^+^·Mes^−^ using: (*a*) laboratory data and (*b*) synchrotron radiation data.

Table 1 ▸ shows the crystal data, experimental parameters and the refinement parameters obtained with both datasets. The comparison between two molecules of the refined structure and two molecules of the DFT-D minimized structure, using the packing similarity feature implemented in *Mercury* (Macrae *et al.*, 2020 ▸) led to an RMSCD of 0.161 Å. This value is lower than 0.35 Å (van de Streek & Neumann, 2014 ▸), indicating that the structure determined is correct. Fig. 4 ▸ shows a comparison of the determined structure with both datasets and the DFT-D optimized structure.

### 4.3. Molecular structure

**Figure 5:**
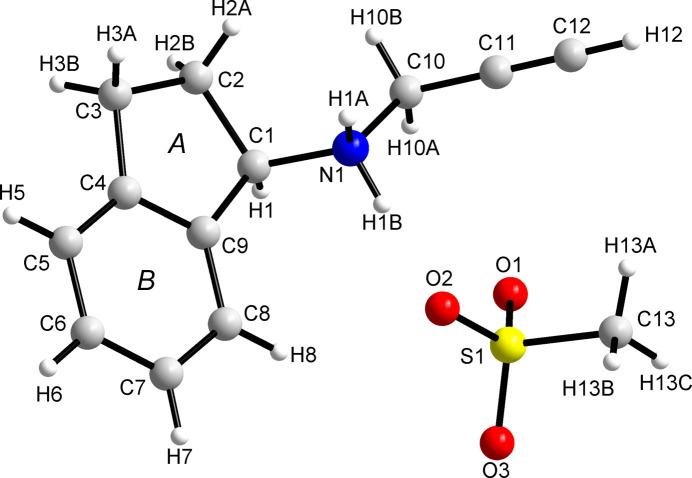
The molecular structure of (*R*)-RasH^+^·Mes^−^ (synchrotron data) showing the labelling scheme for atoms and rings.

Structural features of (*R*)-RasH^+^·Mes^−^ will be discussed using the results of the synchrotron radiation data. Table S1 contains selected bond lengths and bond angles, and torsion angles. Fig. 5 ▸, drawn with *DIAMOND* (Brandenburg, 1999 ▸), depicts the molecular structure of rasagiline mesylate showing the atom and ring labelling scheme.

As mentioned before, the five-membered ring (ring *A*) is planar, in contrast to the structure of NOJKON where the ring has an envelope conformation at C2. The planar ring is reproduced by the DFT calculations and is, therefore, not the result of the averaging of two positions of a disordered C2 atom but is a genuine difference between the molecular geometries in the two crystal structures. Rings *A* and *B* make an angle of 1.61 (12)° while the corresponding angle in NOJKON is 7.79 (16)°. The torsion angles C2—C1—N1—C10 and C9—C1—N1—C10 in (*R*)-RasH^+^·Mes^−^ are −71.0 (2)° and 172.75 (18)°, respectively, and those in NOJKON are −177.2 (3)° and 68.3 (3)°, respectively. The propargyl group makes an angle of 53.3 (2)° with the best plane involving rings *A* and *B*. In contrast, in NOJKON, the corresponding angle is 62.8 (2)°. The bond length C11≡C12 is 1.180 (4) Å and the angle C10—C11≡C12 is 177.7 (3)° which are within the values expected for this group. A recent analysis carried out on two related *N*-propargyl compounds (Güiza *et al.*, 2022 ▸), compared with 30 reports of similar compounds contained in the CSD, indicates that the average C≡C distance is 1.175 Å and the average C—C≡C angle is 177.43°. In NOJKON the C≡C bond length is 1.164 (4) Å and the C—C≡C bond angle is 177.9 (3)°.

### 4.4. Hydrogen bonding

**Figure 6:**
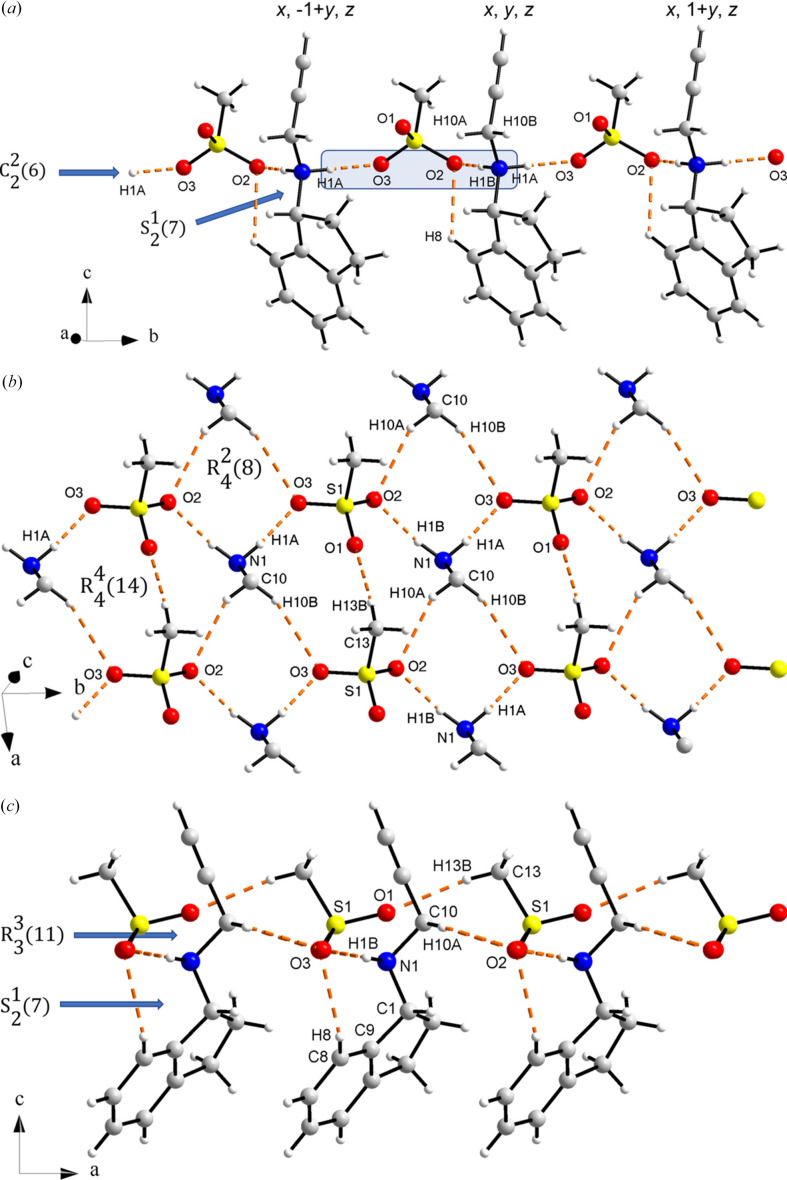
(*a*) Chains of 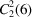 hydrogen bonds along the *b* axis and 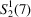 intramolecular hydrogen bonds. (*b*) A layer parallel to the *ab* plane formed by hydrogen bonds with graph set symbols 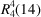 and 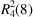 . For clarity, only the atoms of the rasagiline moiety involved in hydrogen bonding are shown. (*c*) Side view of the layer in (*b*) showing the 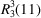 and 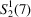 hydrogen bonds above and below the plane.

In the structure of (*R*)-rasagiline mesylate, the oxygen atoms of the mesylate moiety provide the framework for the hydrogen bond network. As can be seen in Table 2 ▸, the –NH_2_ hydrogen atoms participate in strong hydrogen bonds with atoms O2 and O3 of the mesylate anion producing a chain along the *b* axis [Fig. 6 ▸(*a*)]. The sequence of hydrogen bonds can be described by the graph set symbol 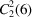 (Etter *et al.*, 1990 ▸; Bernstein *et al.*, 1995 ▸). At the same time, atom O2 forms a cyclic hydrogen bond with one amine hydrogen and one phenyl hydrogen of an (*R*)-RasH^+^ moiety [see Fig. 6 ▸(*a*)] with the same symmetry operation, represented by the symbol 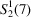 . Furthermore, H10*A*—C10—H10*B* participates in hydrogen bonds with O2 from a molecule with symmetry operation 1 + *x*, *y*, *z* and an O3 with symmetry operation 1 + *x*, 1 + *y*, *z* as depicted in Fig. 6 ▸(*b*). These interactions give rise to layers of hydrogen bonds parallel to the *ab* plane formed by sequences of 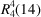 and 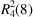 motifs [Fig. 6 ▸(*b*)]. Atom O1 participates in a hydrogen bond with the methyl group of the mesylate anion to form rings represented by the symbol 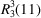 perpendicular to the layers described before [Fig. 6 ▸(*c*)]. It is worth noting that the propyne hydrogen atom is not involved in hydrogen bonding in contrast to other propargyl-containing compounds (Güiza *et al.*, 2022 ▸).

### 4.5. C—H⋯π interactions

**Figure 7:**
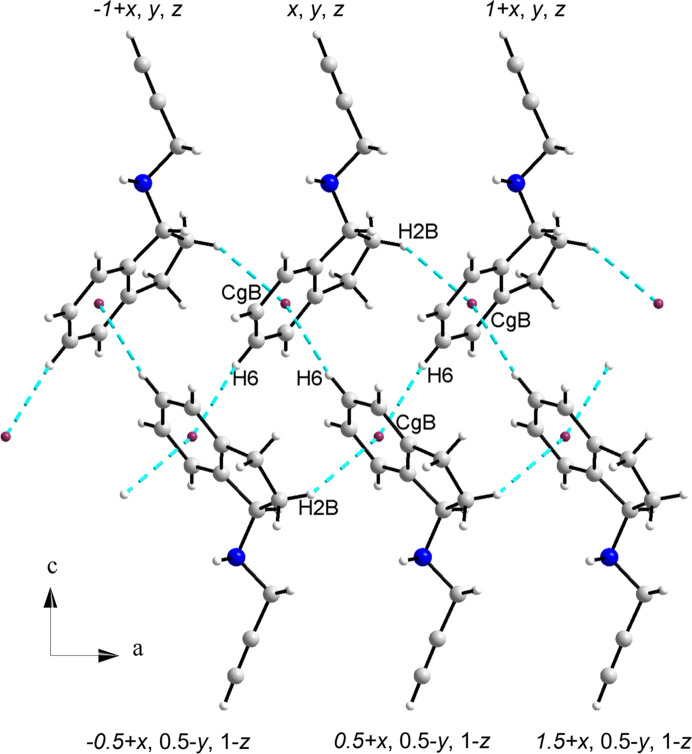
C—H⋯π interactions in the structure of(*R*)-RasH ^+^·Mes^−^.

Two important C—H⋯π interactions are observed in this structure. They are depicted in Fig. 7 ▸ and their geometric parameters, calculated with *PLATON* (Spek, 2020 ▸) are summarized in Table 3 ▸. One contact is relatively strong (2.965 Å) and occurs between C2—H2*B* and the centroid of a ring *B* (Cg*B*) related by translation along the *a* axis. A weaker interaction occurs between C6—H6 and ring *B* of molecules related by a 2_1_ screw axis.

The Aromatics Analyser feature implemented in *Mercury* (Macrae *et al.*, 2020 ▸) suggests a strong interaction between molecules related by a 2_1_ screw axis [5.1760 (14) Å]. However, these molecules are oriented almost perpendicular to each other at 87.68 (12)°. Molecules related by translation along the *a* axis interact via contacts of moderate strength with a centroid–centroid distance of 5.4876 (15) Å but the calculated slippage of the centroids is 3.958 Å, rendering them as weak interactions. Therefore, π⋯π contacts are not considered of importance. In addition, C—H⋯π(C≡C) contacts are not present, in contrast to the structure of NOJKON and of other propargyl-containing compounds, where C—H⋯π(C≡C) short contacts are observed and play an important role in the packing arrangement of the structure.

### 4.6. Packing arrangement

**Figure 8:**
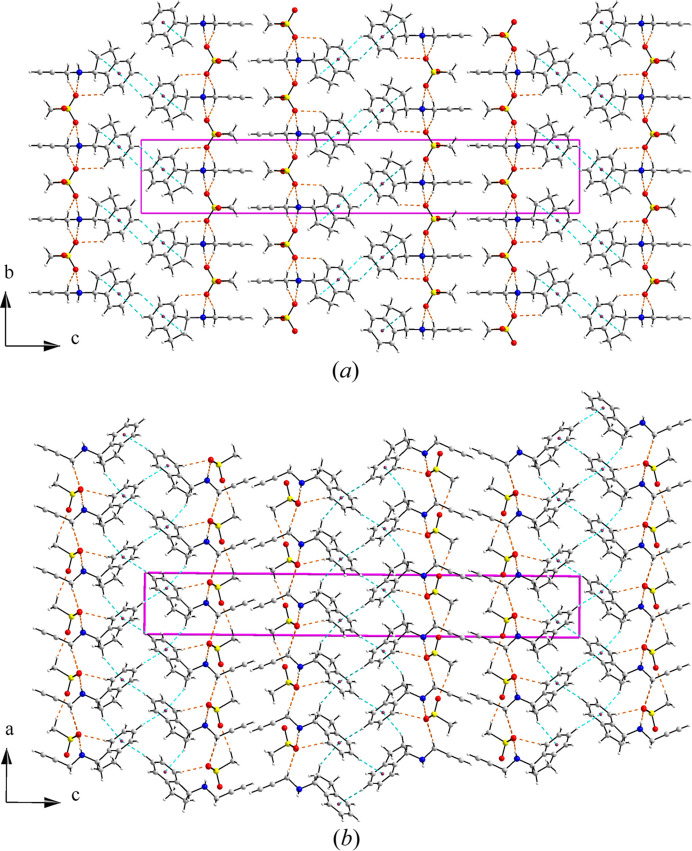
Packing arrangement of (*R*)-RasH^+^·Mes^−^: (*a*) along the *a* axis and (*b*) along the *b* axis. Hydrogen bonds are shown as dashed orange lines and C—H⋯π contacts as dashed cyan lines. The centroids of the six-membered rings are depicted as dark-red dots.

**Figure 9:**
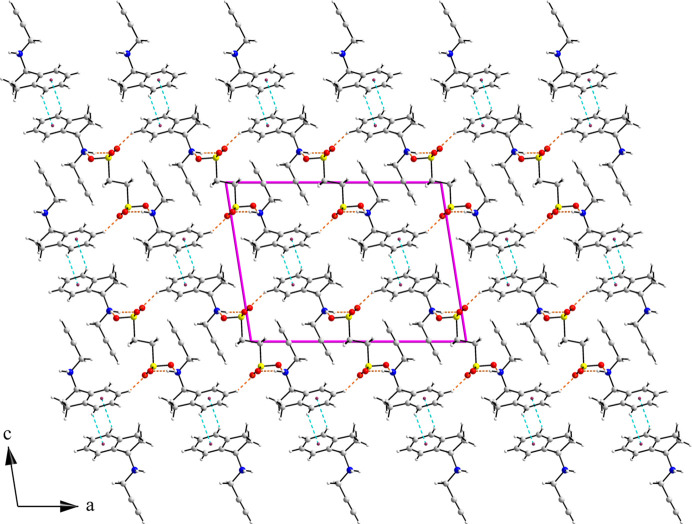
Packing arrangement viewed down the *b* axis of rasagiline ethane­disulfonate (CSD reference code NOJKON).

Based on the patterns of hydrogen bonding and C—H⋯π interactions observed in (*R*)-RasH ^+^·Mes^−^ the structure can be described as layers (parallel to the *ab* plane) resulting from hydrogen bonding between the mesylate oxygen atoms and the amine hydrogen atoms, with the propargyl and indenamine groups distributed above and below the plane. The layers then form a double layer by C—H⋯π interactions between the indenamine groups of molecules related by a 2_1_ screw axis. The double layers stack along the *c* axis via van der Waals interactions. Fig. 8 ▸ shows the views down the *a* and *b* axes of the packing arrangement in (*R*)-RasH^+^·Mes^−^. In the structure of NOJKON, the two sulfonate ends of the anion provide the connection between the rasagiline moieties to form columns which repeat along the *c* axis joined by C—H⋯π contacts (Fig. 9 ▸).

### 4.7. Hirshfeld surface analysis

**Figure 10:**
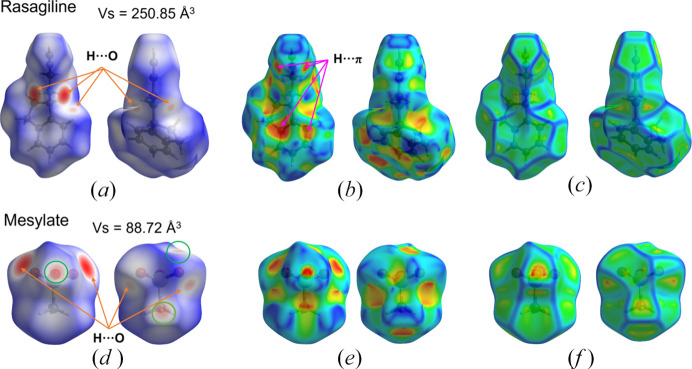
Hirshfeld surface mapped onto (*a*) *d*_norm_, (*b*) shape index and (*c*) curvedness for the (*R*)-RasH^+^ moiety, and (*d*), (*e*), (*f*), respectively, for the Mes^−^ moiety.

**Figure 11:**
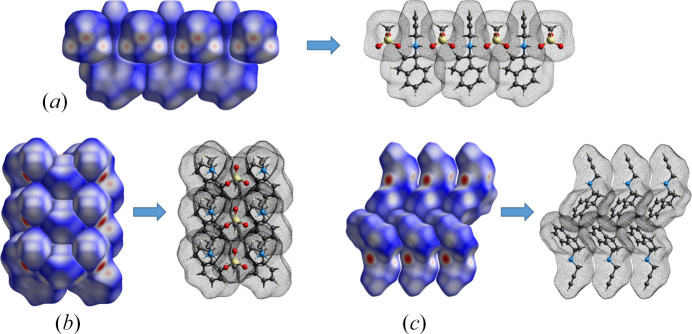
Packing of molecules due to H⋯O hydrogen bonds (*a*,*b*) and H⋯π interactions (*c*).

The Hirshfeld surface mapped onto *d*_norm_, shape index, and curvedness for rasagiline mesylate are shown in Fig. 10 ▸. The volume of the surface within the unit cell is 1358.28 Å^3^, corresponding to 97.4% of the unit-cell volume. This indicates a compact packing of the molecules and almost no void space. O⋯H short-range contacts are observed as red spots on the surface of (*R*)-RasH^+^ moieties [yellow arrows, Fig. 10 ▸(*a*)] and are complemented by the spots observed on the surface of the Mes^−^ ion [yellow arrows, Fig. 10 ▸(*d*)]. In addition, intermolecular O⋯H interactions between Mes^−^ counterions appear as three faint red spots [green circles, Fig. 10 ▸(*d*)]. These interactions result in chains of (*R*)-RasH^+^ units which alternate with chains of Mes^−^ counterions along the *a* and *b* axes, as depicted in Fig. 11 ▸.

The Hirshfeld surface mapped onto the shape index [Fig. 10 ▸(*b*)] shows the typical red areas associated with H⋯π interactions. These interactions occur between H atoms of rings *A* and *B* (defined in Fig. 5 ▸) with the centroid of ring *B*. These interactions and the interactions in which the propargyl end participate are evident in the curvedness map as flat areas [Fig. 10 ▸(*c*)]. Fig. 11 ▸(*c*) shows how these groups pack. The curvedness representation of the Mes^−^ moiety results in flat areas where the methyl group is located [Fig. 10 ▸(*f*)] which complement the flat areas of the propargyl group. Similar patterns are observed for the rasagiline moiety in rasagiline ethane­disulfonate (NOJKON) as shown in Fig. S1.

**Figure 12:**
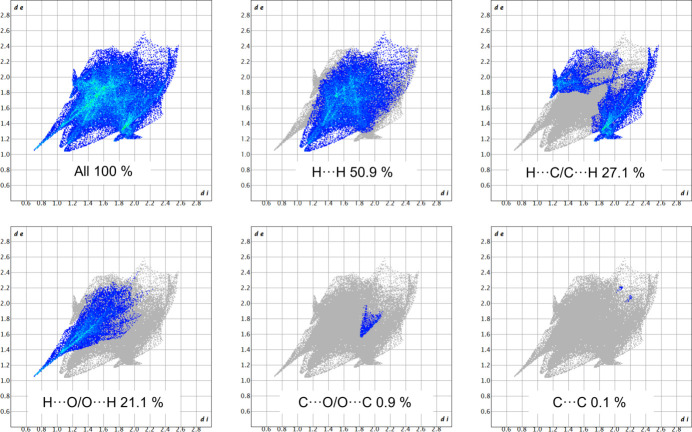
Fingerprint plots for the (*R*)-RasH^+^ moiety in (*R*)-RasH^+^·Mes^−^ and % contributions from specific pairs of interatomic interactions.

**Figure 13:**
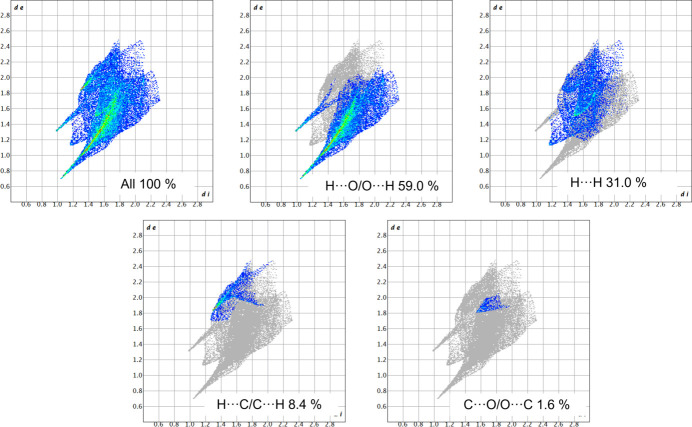
Fingerprint plots for the Mes^−^ moiety in (*R*)-RasH ^+^·Mes^−^ and % contributions from specific pairs of interatomic interactions.

Fingerprint plots for (*R*)-RasH^+^ and Mes^−^ units are presented in Figs. 12 ▸ and 13 ▸. The most important interactions for (*R*)-RasH^+^ (Fig. 12 ▸) are the H⋯H contacts which contribute 50.9%, followed by H⋯C/C⋯H interactions (27.1%) and H⋯O/O⋯H contacts (21.1%). The C⋯O/O⋯C and C⋯C interactions contribute 0.9 and 0.1%, respectively. For the Mes^−^ anion, the H⋯O/O⋯H contacts contribute 59.0%, H⋯H 31.0%, H⋯C/C⋯H 8.4% and C⋯O/O⋯C 1.6% (Fig. 13 ▸). The fingerprint plots for the complete (*R*)-RasH^+^·Mes^−^ unit in (*R*)-rasagiline mesylate are shown in Fig. S2. The contribution of the H⋯H contacts remains the same (50.9%) but the H⋯O/O⋯H contacts now represent 26.1% while the H⋯C/C⋯H interactions contribute 22.3%.

In the structure of NOJKON the contributions of the different interactions in which the rasagiline moiety is involved are very similar: 50.9% for H⋯H, 28.4% for H⋯C/C⋯H, 20.8% for H⋯O/O⋯H, 0.4% for C⋯O/O⋯C and <0.1% for C⋯C. These plots are shown in Fig. S3.

### 4.8. Spectroscopic analysis

**Figure 14:**
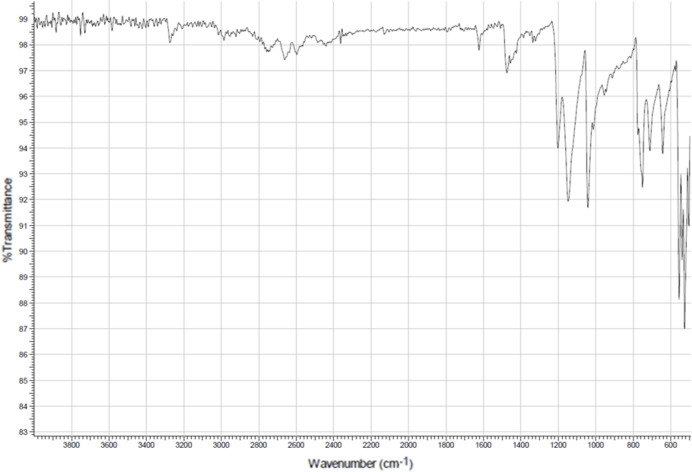
ATR-IR spectra of (*R*)-RasH^+^·Mes^−^.

The ATR-IR spectrum of (*R*)-RasH^+^·Mes^−^ (Fig. 14 ▸) shows the characteristic absorption bands of the functional groups present in the compound. The bands corresponding to the stretching of the N—H bonds of the secondary amine at 3275 cm^−1^ overlapping the C*sp*—H band are clearly observed. The aromatic C*sp* ^2^—H appears at 3010 cm^−1^ and the aliphatic C*sp* ^3^—H appears at 2986 cm^−1^. Between 2220 and 2200 cm^−1^ a weak band corresponding to the C≡C stretching vibration is observed, while the aromatic C=C band appears at 1624 cm^−1^. As mentioned before, upon exposure to synchrotron radiation, the irradiated portions of the sample turned dark. The spectrum of these portions showed a decrease in the relative intensities of some absorptions but no other changes that could indicate severe decomposition or reaction of the material.

### 4.9. Thermal analysis

**Figure 15:**
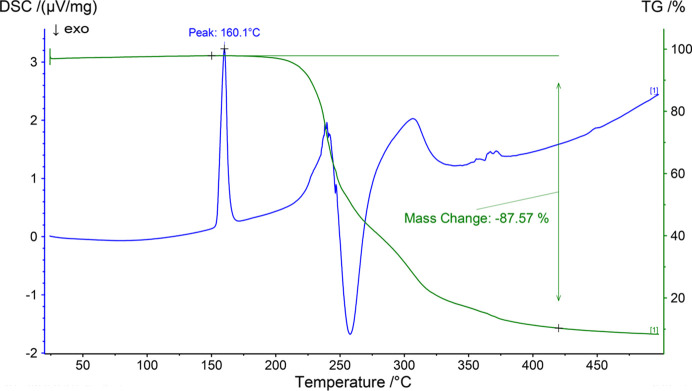
TGA-DSC analysis of (*R*)-RasH^+^·Mes^−^.

The TGA curve (Fig. 15 ▸) shows that the material is stable up to 150°C. The thermogram presents several thermal events of mass loss between 100 and 420°C that integrate for a total of 87.27%. On the other hand, the DSC curve shows an endothermic transition between 150 and 170°C with peak temperature *T*_p_ = 160.1°C corresponding to the melting of the compound. The transitions observed above 200°C are associated with the mass losses observed in the TGA analysis and are attributed to the decomposition processes of the compound.

## 5. Conclusions

The structure of (*R*)-rasagiline mesylate [(*R*)-RasH^+^·Mes^−^], a MAO-B inhibitor used in the treatment of Parkinson’s disease, was determined from laboratory and synchrotron powder diffraction data. The structure was determined satisfactorily using the laboratory data. The refinement of the March–Dollase parameter for preferred orientation was improved with the synchrotron data. In (*R*)-RasH^+^·Mes^−^, rings *A* and *B* (Fig. 5 ▸) of the (*R*)-RasH^+^ moiety are planar, in contrast with the previously reported structure of rasagiline ethane­disulfonate (CSD reference code NOJKON) where the five-membered ring (*A*) has an envelope conformation at C2. The Mes^−^ anions are distributed in layers parallel to the *ab* plane and connect to (*R*)-RasH^+^ cations via extensive hydrogen bonding. Their aromatic rings (*B*) interact with other *B* rings via C—H⋯π interactions on one side of the plane formed by the Mes^−^ ions while on the other side of the plane, the propargyl groups of (*R*)-RasH^+^ and the methyl groups of the Mes^−^ units alternate along the *a* axis and face each other along the *c* axis. Hirshfeld surface analysis indicates that the main contributions to the packing of (*R*)-RasH^+^ moieties are from H⋯H (50.9%), followed by H⋯C/C⋯H (27.1%) and H⋯O/O⋯H (21.1%) interactions. The corresponding percent contributions in the reported ethane­disulfonate (NOJKON) are similar (50.3%, 28.4% and 20.8%, respectively).

## 6. Supplementary material

The supplementary material includes a Crystallographic Information File (CIF) containing the results of the Rietveld refinements and the DFT-D geometry optimization results. The laboratory powder diffraction data has been deposited with the International Centre for Diffraction Data (ICDD) for inclusion in the Powder Diffraction File (PDF 00-073-1393).

Crystal structure: contains datablock(s) I, I. DOI: 10.1107/S2052520623007758/ra5135sup1.cif

Structure factors: contains datablock(s) I. DOI: 10.1107/S2052520623007758/ra5135Isup2.hkl

Rietveld powder data: contains datablock(s) I. DOI: 10.1107/S2052520623007758/ra5135Isup3.rtv

DFT-D Results. DOI: 10.1107/S2052520623007758/ra5135sup4.txt

Table S1 and Figs S1-S3. DOI: 10.1107/S2052520623007758/ra5135sup5.pdf

CCDC reference: 2293343

## Figures and Tables

**Table 1:** Crystal data, experimental parameters, and refinement results for (*R*)-RasH^+^·Mes^−^ For all datasets: C_13_H_17_NO_3_S, *M*_r_ = 267.34, orthorhombic, *P*2_1_2_1_2_1_, *Z* = 4, melting point 433.1 K. The specimen was prepared at 298 K and 100 kPa. Experiments were carried out at 298 K. Refinement was with 99 restraints and with four constraints. Only non-H-atom coordinates were refined. The profile function was pseudo-Voigt.

	Cu *K*α_1_, λ = 1.5418 Å	Synchrotron, λ = 0.354176 Å
Crystal data		
*a*, *b*, *c* (Å)	5.4913 (3), 6.5358 (4), 38.9501 (16)	5.48753 (1), 6.528939 (12), 38.94313 (9)
*V* (Å^3^)	1397.93 (13)	1395.25 (1)
μ (mm^−1^)	2.07	0.05
Particle morphology	Fine powder	Fine powder
Colour	White	White
Specimen shape, size (mm)	Flat sheet, 24.5 × 24.5	Cylinder, 15 × 1.0

Data collection		
Diffractometer	Bruker D8 ADVANCE	ESRF powder diffractometer, beamline ID22
Radiation source	Sealed X-ray tube	Synchrotron
Monochromator	None	Si 111 double crystal
Specimen mounting	Flat plate low-background Si single crystal specimen holder	1.0 mm thin-walled borosilicate capillary
Data collection mode	Reflection	Transmission
Scan method	Step	Continuous, 20° min^−1^
2θ min, max, step values (°)	4.00, 50.00, 0.02	0.50, 20.00, 0.0005

Refinement		
March–Dollase preferred orientation parameter	0.642 (1)	1.140 (1)
*R*_p_, *R*_wp_, *R*_exp_, *R*_Bragg_	0.061, 0.084, 0.065, 0.022	0.076, 0.081, 0.010, 0.028
Goodness of fit, χ^2^	1.680	7.814
No. of parameters	156	125
(Δ/σ)_max_	0.001	0.001

Computer programs: *DIFFRAC.SUITE* (Bruker, 2011 ▸), *TOPAS-Academic* (Coelho, 2018 ▸), *DASH* (David *et al.*, 2006 ▸), *DIAMOND* (Brandenburg, 1999 ▸), *Mercury* (Macrae *et al.*, 2020 ▸), *enCIFer* (Allen *et al.*, 2004 ▸), *publCIF* (Westrip, 2010 ▸).

**Table 2:** Hydrogen bond parameters for (*R*)-RasH^+^·Mes^−^

*D*—H⋯*A*	*D*—H (Å)	H⋯*A* (Å)	*D*⋯*A* (Å)	*D*—H⋯*A* (°)
N1—H1*A*⋯O3^i^	0.953 (6)	1.815 (6)	2.764 (3)	173.3 (5)
N1—H1*B*⋯O2	0.953 (6)	1.853 (6)	2.798 (4)	171.7 (5)
C8—H8⋯O2	0.952 (6)	2.497 (6)	3.180 (4)	128.7 (4)
C10—H10*A* ^ii^⋯O2	0.951 (6)	2.486 (6)	3.366 (4)	153.9 (5)
C10—H10*B* ^iii^⋯O3	0.953 (6)	2.562 (6)	3.469 (3)	159.2 (5)
C13—H13*B* ^iv^⋯O1	0.951 (5)	2.435 (5)	3.379 (3)	171.6 (5)

Symmetry operations: (i) *x*, 1 + *y*, *z*; (ii) 1 + *x*, *y*, *z*; (iii) 1 + *x*, 1 + *y*, *z*; (iv) −1 + *x*, *y*, *z*.

**Table 3:** Geometry of C—H⋯π(ring) interactions in (*R*)-RasH^+^·Mes^−^ calculated with *PLATON* (Spek, 2020 ▸)

*X*—H⋯π	H⋯*Cg* (Å)	H–Perp (Å)	γ (°)	*X*—H⋯*Cg*(*B*) (°)	*X*⋯*Cg*(*B*) (Å)	*X*—H⋯π (°)
C2—H2*B*⋯*Cg*(*B*)^ii^	2.965 (6)	2.96	1.65	144.5 (5)	3.777 (2)	56.00
C6—H6⋯*Cg*(*B*)^v^	3.209 (6)	3.000 Å	20.79	138.2 (4)	3.970 (3)	60.10

Symmetry operations: (ii) 1 + *x*, *y*, *z*; (v): −½ + *x*, ½ − *y*, 1 − *z*. γ is the angle between the *Cg*(*B*)—H vector and normal to ring *B*.
