# Supplementary material for: Hydrogen bonding patterns and C—H⋯π inter­actions in the structure of the antiparkinsonian drug (R)-rasagiline mesylate determined using laboratory and synchrotron X-ray powder diffraction data
Source: Acta Crystallogr B Struct Sci Cryst Eng Mater. 2023 Oct 11;79(Pt 6):462–72. doi: 10.1107/S2052520623007758 (PMC10833353; doi:10.1107/S2052520623007758)
Supplement: Supplementary file 5 [file b-79-00462-sup5.pdf]

STRUCTURAL SCIENCE
CRYSTAL ENGINEERING
MATERIALS

Volume 79 (2023)

Supporting information for article:

Hydrogen bonding patterns and C–H···π interactions in the structure of the antiparkinsonian drug (*R*)-Rasagiline Mesylate determined using laboratory and synchrotron X-ray powder diffraction data

Analio J. Dugarte-Dugarte, Robert A. Toro, Jacco van de Streek, José Antonio Henao, Andrew N. Fitch, Catherine Dejoie, José Miguel Delgado and Graciela Díaz de Delgado

Table S1

Selected geometric parameters (Å, º) for (*R*)-RasH^{+}·Mes^{-}.

S1—C13	1.751 (3)	C10—C11	1.443 (3)
S1—O3	1.451 (3)	C11—C12	1.180 (4)
S1—O1	1.453 (3)	C1—H1	0.945 (5)
S1—O2	1.453 (3)	C2—H2A	0.948 (5)
N1—C1	1.517 (2)	C2—H2	0.948 (5)
N1—C10	1.501 (2)	C3—H3A	0.957 (5)
C1—C9	1.510 (2)	C3—H3B	0.953 (6)
N1—H1B	0.953 (6)	C5—H5	0.951 (6)
C1—C2	1.5489 (10)	C6—H6	0.952 (6)
N1—H1A	0.953 (6)	C7—H7	0.955 (6)
C2—C3	1.526 (3)	C8—H8	0.952 (6)
C3—C4	1.498 (4)	C10—H10B	0.953 (6)
C4—C9	1.379 (3)	C10—H10A	0.951 (6)
C4—C5	1.391 (4)	C12—H12	0.950 (6)
C5—C6	1.383 (4)	C13—H13A	0.955 (6)
C6—C7	1.388 (3)	C13—H13B	0.951 (5)
C7—C8	1.392 (4)	C13—H13C	0.953 (5)
C8—C9	1.397 (3)		
O2—S1—O3	111.88 (18)	N1—C1—H1	106.1 (4)
O2—S1—C13	105.54 (15)	C1—C2—H2A	111.1 (4)
O3—S1—C13	105.37 (15)	C1—C2—H2	110.3 (4)
O1—S1—C13	107.53 (14)	H2—C2—H2A	106.2 (5)
O1—S1—O2	113.16 (18)	C3—C2—H2	111.1 (4)
O1—S1—O3	112.69 (18)	C3—C2—H2A	110.6 (3)

C1—N1—C10	112.75 (13)	C4—C3—H3B	111.5 (4)
C2—C1—C9	104.68 (12)	C2—C3—H3A	111.8 (4)
N1—C1—C2	110.67 (13)	C2—C3—H3B	111.0 (4)
N1—C1—C9	111.72 (11)	C4—C3—H3A	111.2 (4)
C1—N1—H1B	110.9 (4)	H3A—C3—H3B	106.7 (6)
C1—N1—H1A	109.3 (4)	C4—C5—H5	120.5 (5)
C10—N1—H1B	108.3 (4)	C6—C5—H5	120.5 (5)
H1A—N1—H1B	105.6 (5)	C7—C6—H6	118.7 (4)
C10—N1—H1A	109.7 (4)	C5—C6—H6	119.9 (4)
C1—C2—C3	107.59 (13)	C6—C7—H7	120.0 (4)
C2—C3—C4	104.66 (19)	C8—C7—H7	120.4 (4)
C5—C4—C9	120.1 (2)	C7—C8—H8	120.1 (4)
C3—C4—C9	112.5 (2)	C9—C8—H8	121.1 (4)
C3—C4—C5	127.5 (2)	N1—C10—H10B	108.1 (4)
C4—C5—C6	119.0 (2)	N1—C10—H10A	107.8 (4)
C5—C6—C7	121.4 (3)	H10A—C10—H10B	107.9 (5)
C6—C7—C8	119.6 (2)	C11—C10—H10A	111.1 (4)
C7—C8—C9	118.81 (19)	C11—C10—H10B	110.0 (4)
C1—C9—C4	110.44 (17)	C11—C12—H12	178.9 (6)
C1—C9—C8	128.39 (16)	S1—C13—H13A	107.8 (4)
C4—C9—C8	121.08 (19)	S1—C13—H13B	106.9 (4)
N1—C10—C11	111.75 (18)	S1—C13—H13C	108.0 (4)
C10—C11—C12	177.7 (3)	H13A—C13—H13B	112.0 (6)
C2—C1—H1	112.2 (3)	H13A—C13—H13C	110.7 (5)
C9—C1—H1	111.6 (4)	H13B—C13—H13C	111.4 (6)

C10—N1—C1—C2	−71.0 (2)	C3—C4—C5—C6	−178.4 (3)

C10—N1—C1—C9	172.75 (18)	C3—C4—C9—C1	−3.8 (3)
C1—N1—C10—C11	177.5 (2)	C3—C4—C9—C8	179.4 (2)
N1—C1—C2—C3	−119.23 (16)	C9—C4—C5—C6	−0.7 (4)
C9—C1—C2—C3	1.28 (16)	C5—C4—C9—C8	1.4 (4)
N1—C1—C9—C8	−62.3 (2)	C5—C4—C9—C1	178.2 (2)
C2—C1—C9—C4	1.44 (19)	C4—C5—C6—C7	0.3 (4)
N1—C1—C9—C4	121.24 (18)	C5—C6—C7—C8	−0.6 (4)
C2—C1—C9—C8	177.95 (19)	C6—C7—C8—C9	1.3 (4)
C1—C2—C3—C4	−3.3 (2)	C7—C8—C9—C1	−177.85 (19)
C2—C3—C4—C9	4.4 (3)	C7—C8—C9—C4	−1.7 (3)
C2—C3—C4—C5	−177.7 (3)		

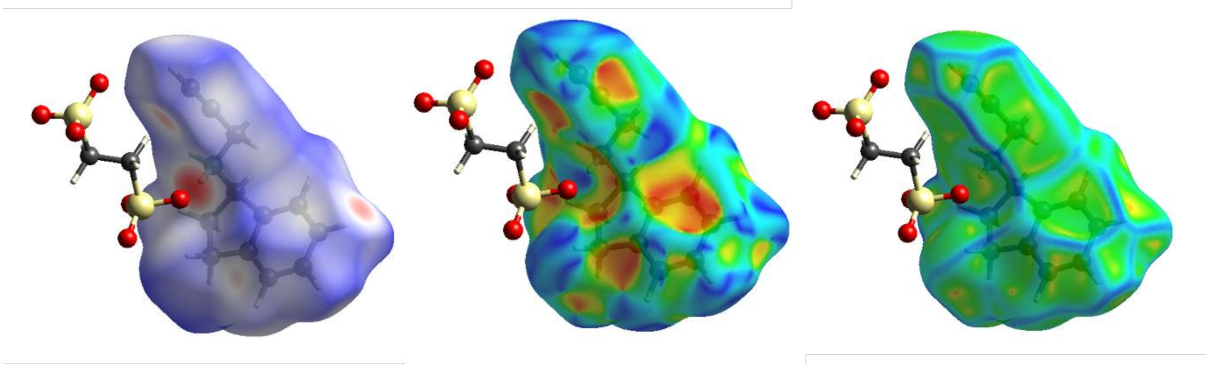

Figure S1 Hirshfeld surface mapped onto (a) d_{norm}, (b) shape index, and (c) curvedness for the (*R*)-RasH^{+} moiety in (*R*)-rasagiline ethanedisulfonate, Refcode NOJKON (Brüning et al., 2008).

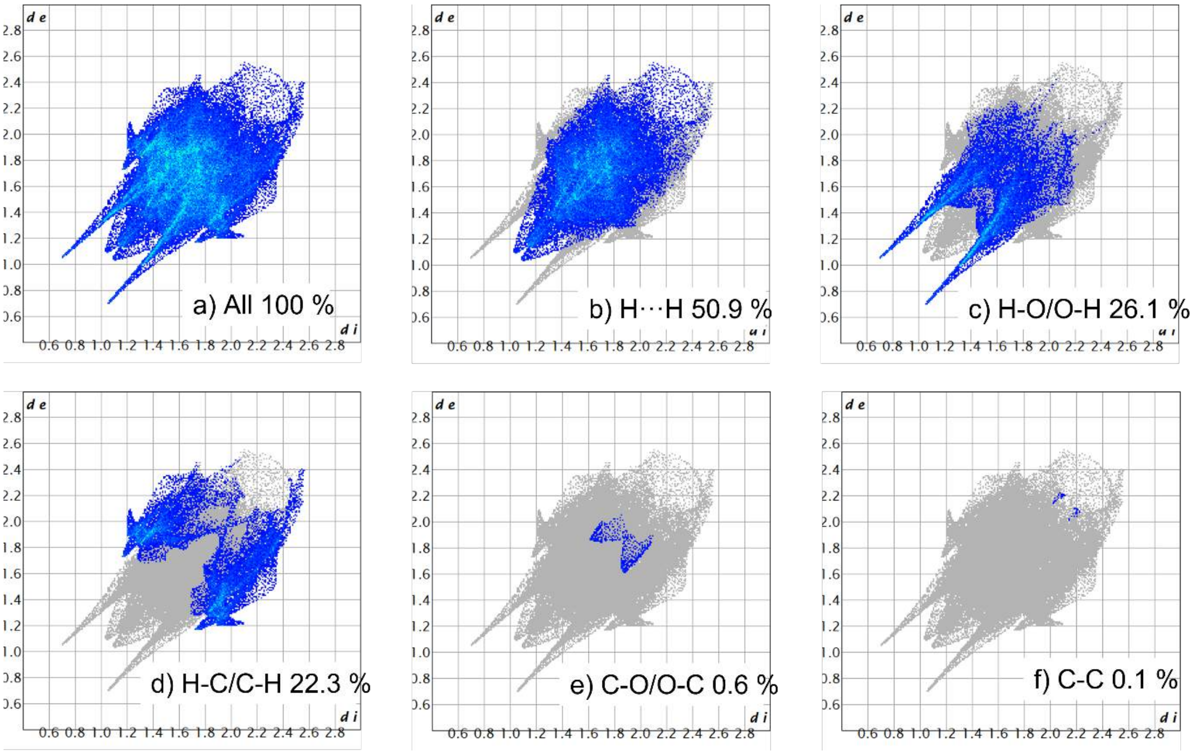

Figure S2 Fingerprint plots for the complete (*R*)-$RasH^{+}\cdot Mes^{-}$ unit in (*R*)-rasagiline mesylate and combined % contributions from specific pairs of interatomic interactions.

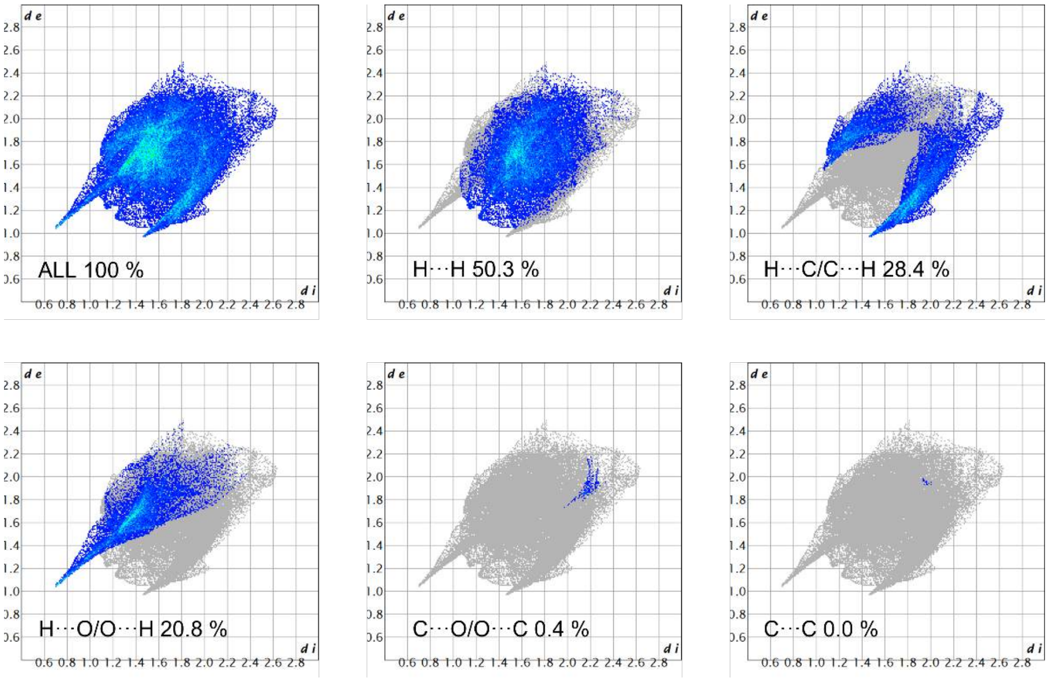

Figure S3 Fingerprint plots for the (*R*)-RasH+ moiety in (R)-rasagiline ethanedisulfonate, Refcode NOJKON (Brüning et al., 2008) and % contributions from specific pairs of interatomic interactions.
